# The 31-Gene Expression Profile Test Outperforms AJCC in Stratifying Risk of Recurrence in Patients with Stage I Cutaneous Melanoma

**DOI:** 10.3390/cancers16020287

**Published:** 2024-01-09

**Authors:** Sebastian Podlipnik, Brian J. Martin, Sonia K. Morgan-Linnell, Christine N. Bailey, Jennifer J. Siegel, Valentina I. Petkov, Susana Puig

**Affiliations:** 1 Dermatology Department, IDIBAPS, Hospital Clínic de Barcelona, Universitat de Barcelona, 08036 Barcelona, Spain; 2 Castle Biosciences, Inc., Friendswood, TX 77546, USA; 3 Surveillance Research Program, National Cancer Institute, Bethesda, MD 20892, USA; valentina.petkov@nih.gov

**Keywords:** 31-gene expression profile, cutaneous melanoma, AJCC, prognosis, staging, gene expression profiling

## Abstract

**Simple Summary:**

Clinicians currently estimate the risk of a patient’s melanoma returning or spreading using tumor thickness and other characteristics. Most patients are diagnosed with stage I disease and are considered to have a low risk of a poor outcome, but they account for the largest number of melanoma deaths each year. The 31-gene expression profile test (31-GEP) looks at the molecular biology of the tumor to determine a patient’s risk of cancer returning or spreading. In this study, the 31-GEP was better at predicting cancer progression than current melanoma staging. The 31-GEP can help doctors personalize care and make better treatment and management plans for patients.

**Abstract:**

Background: Patients with stage I cutaneous melanoma (CM) are considered at low risk for metastasis or melanoma specific death; however, because the majority of patients are diagnosed with stage I disease, they represent the largest number of melanoma deaths annually. The 31-gene expression profile (31-GEP) test has been prospectively validated to provide prognostic information independent of staging, classifying patients as low (Class 1A), intermediate (Class 1B/2A), or high (Class 2B) risk of poor outcomes. Methods: Patients enrolled in previous studies of the 31-GEP were combined and evaluated for recurrence-free (RFS) and melanoma-specific survival (MSS) (n = 1261, “combined”). A second large, unselected real-world cohort (n = 5651) comprising clinically tested patients diagnosed 2013–2018 who were linked to outcomes data from the NCI Surveillance, Epidemiology, and End Results (SEER) Program registries was evaluated for MSS. Results: Combined cohort Class 1A patients had significantly higher RFS than Class 1B/2A or Class 2B patients (97.3%, 88.6%, 77.3%, *p* < 0.001)—better risk stratification than AJCC8 stage IA (97.5%) versus IB (89.3%). The SEER cohort showed better MSS stratification by the 31-GEP (Class 1A = 98.0%, Class 1B/2A = 97.5%, Class 2B = 92.3%; *p* < 0.001) than by AJCC8 staging (stage IA = 97.6%, stage IB = 97.9%; *p* < 0.001). Conclusions: The 31-GEP test significantly improved patient risk stratification, independent of AJCC8 staging in patients with stage I CM. The 31-GEP provided greater separation between high- (Class 2B) and low-risk (Class 1A) groups than seen between AJCC stage IA and IB. These data support integrating the 31-GEP into clinical decision making for more risk-aligned management plans.

## 1. Introduction

The American Joint Committee on Cancer 8th edition (AJCC8) stages patients with cutaneous melanoma (CM) based on tumor thickness and ulceration status and whether patients have localized disease, locoregional involvement, or distant metastasis [1]. Stage I CM is considered low risk for recurrence and melanoma-specific mortality [1,2,3]; however, because of the large absolute number of patients diagnosed as stage I, this group accounts for more deaths each year than any other group [4,5]. Further, studies have shown that overall survival rates are not different between stage IA and stage IB patients [6]. As such, the AJCC 8th edition does not provide sufficient prognostic information for patients with stage I CM, suggesting prognostic improvements are needed in this population [6,7]. Moreover, those considered low risk for tumor recurrence can experience high anxiety levels, fear of cancer recurrence, and post-traumatic stress disorder about their diagnosis, even after curative surgery [8,9,10]. Patients state that having additional prognostic information about their tumor diagnosis relieves uncertainty and helps them plan for the future [11]. Moreover, breast cancer patients who receive low-risk results from molecular prognostic testing report lower anxiety levels [12]. Therefore, prognostic tests that can identify patients at high risk of poor outcomes or confirm that patients are truly at low risk of disease progression are needed to supplement current staging criteria for patients with CM and can offer benefits to physicians for patient management planning and may provide psychological benefits to patients.

The 31-gene expression profile test (31-GEP) is validated to provide additional prognostic information on tumor recurrence risk independent of current staging factors [13,14,15,16,17,18,19] by classifying patients as having a low risk (Class 1A), intermediate risk (Class 1B/2A), or high risk (Class 2B) of tumor recurrence, metastasis, and melanoma-specific mortality [18,20,21,22,23,24,25]. Previous studies have found that integrating the 31-GEP with clinical and pathological factors (i31-GEP) improves risk stratification compared to other clinicopathologic-only tools, such as the Melanoma Institute of Australia’s (MIA) online nomogram [26].

This study assessed 5-year recurrence-free survival (RFS) and melanoma-specific survival (MSS) risk stratification among patients with stage I CM using the 31-GEP. The 31-GEP provided greater separation between high- (Class 2B) and low-risk (Class 1A) groups than seen between AJCC stage IA and IB.

## 2. Materials and Methods

### 2.1. Patients

Two cohorts of patients were used in this study. The first was a pooled cohort of patients enrolled in retrospective and prospective studies were analyzed (n = 1261; “combined” cohort) [16,17,18,19,23]. The second cohort included clinically tested patients who were linked to CM cases (2013–2018 diagnosis years) ascertained by central cancer registries participating in the National Cancer Institute’s Surveillance, Epidemiology, and End Results (SEER) Program (“SEER” cohort). Because the SEER data cover over one-third of the United States population, the SEER cohort allows assessment of a large, unselected cohort of patients with stage I CM (n = 5621).

### 2.2. Survival Analysis

Five-year RFS (combined cohort) and MSS (combined and SEER cohorts) were estimated using Kaplan–Meier analysis with the log-rank test. SEER data do not include recurrence or metastasis data after the initial diagnosis; therefore, we could only analyze MSS for this cohort. Recurrence was defined as any regional or distant recurrence. Distant metastasis was defined as tumor metastasis beyond the regional nodal basin. Melanoma-specific survival was defined as the time from the date of diagnosis to the date of death from melanoma.

### 2.3. Statistical Analysis

Multivariable Cox regression analysis was used to compare predictors of recurrence or survival. The likelihood ratio was calculated for each univariable or multivariable Cox model and indicates the degree of predictive power for a given model over a null model (no predictors). Models based on the same cohort and event type can be compared statistically to determine whether a given set of predictors yields a significant improvement in model performance over another set based on the fit of each model to the observed data (log likelihood) using an analysis of deviance test (model ANOVA, alpha = 0.05). The fit of the model to the data is the source of model deviance, which is used in place of variance for the ANOVA test. *p* < 0.05 was considered statistically significant for all comparisons. Cox regression and statistical testing were performed using the R statistical package (v.4.1.2).

## 3. Results

### 3.1. Survival Analysis in the Combined Cohort

**Figure 1:**
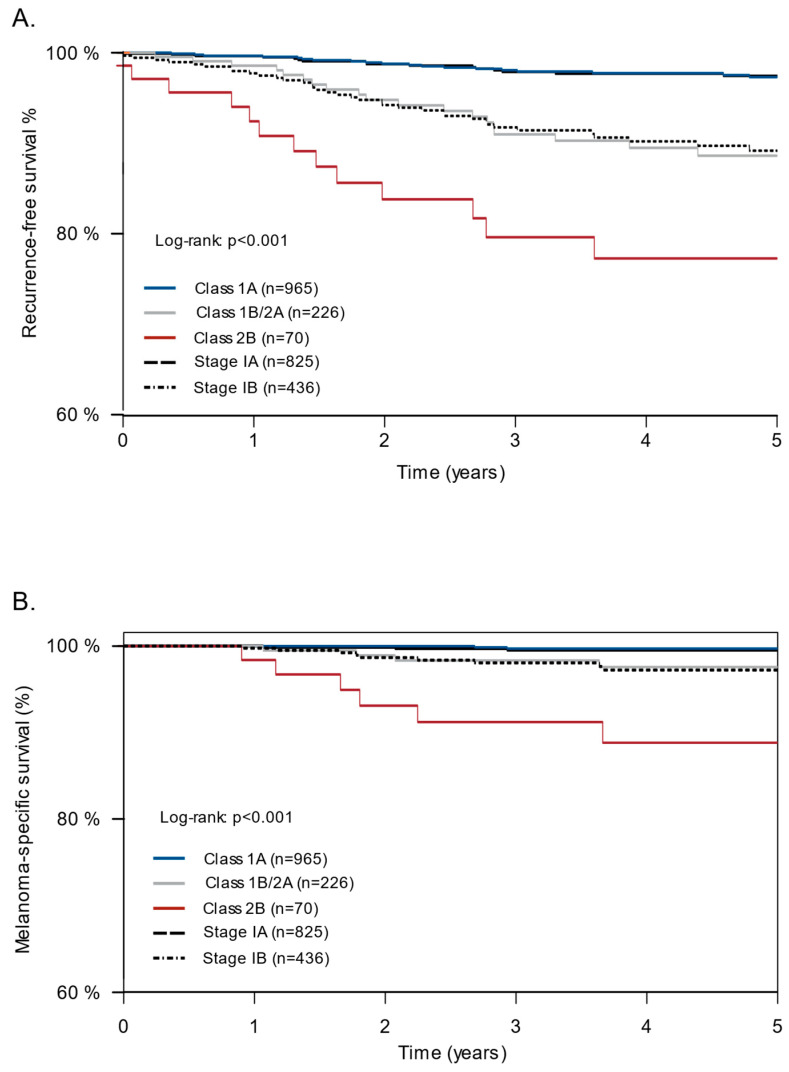
Combined cohort: recurrence-free survival (RFS) and melanoma-specific survival (MSS) risk stratification by the 31-GEP and AJCC8 in stage I tumors. KM curves showing 5-year RFS (**A**) and MSS (**B**) among patients in the combined cohort based on 31-GEP result and AJCC8 stage.

Patient demographics for the combined cohort are shown in Table 1. Patients with stage IA CM had higher 5-year RFS rates than those with stage IB CM (97.5% vs. 89.3%) (Figure 1). Integrating the 31-GEP with AJCC staging improved risk stratification compared with risk stratification by AJCC alone. Patients with a Class 2B 31-GEP result had lower 5-year RFS (77.3%, 95% CI: 66.9–89.2%) than those with a Class 1A (97.3%, 95% CI: 96.1–98.5%) or 1B/2A (88.6%, 95% CI: 83.8–93.7%) 31-GEP result (*p* < 0.001) or than all patients with stage IB CM (Figure 1). The 31-GEP test also provided better stratification of 5-year MSS than did AJCC staging. Patients with a Class 2B result had a lower 5-year MSS (88.8%, 95% CI: 80.6–97.8%) than those with a Class 1A (99.7%, 95% CI: 99.3–100%) or Class 1B/2A (97.6%, 95% CI: 95.2–100%) result. Only slight differences were seen in the 5-year MSS between stage IA (99.5%, 95% CI: 99.0–100%) and stage IB (97.2%, 95% CI: 95.4–99.1%) CM.

Multivariable analysis indicated that the 31-GEP Class 2B result was the strongest predictor of recurrence in stage I CM (HR = 5.16, *p* < 0.001), with Class 1B/2A (HR = 2.63, *p* = 0.002) and stage IB (HR = 2.98, *p* < 0.001) also significant predictors of recurrence. Similar results were observed for 5-year MSS; however, in this cohort, the only significant predictor of MSS was 31-GEP Class 2B (HR = 11.08, *p* < 0.001) (Table 2).

Next, two regression models were built to assess the contribution of the 31-GEP to AJCC staging for RFS risk prediction. Adding 31-GEP to AJCC staging significantly increased the log likelihood, indicating that the model combining 31-GEP with AJCC staging explained the data better than the model with AJCC staging alone (X^2^ = 20.9, *p* < 0.001) (Table 3). Thus, adding 31-GEP testing to AJCC staging improved RFS risk prediction compared to using AJCC staging alone.

### 3.2. Survival Analysis in the SEER Cohort

**Figure 2:**
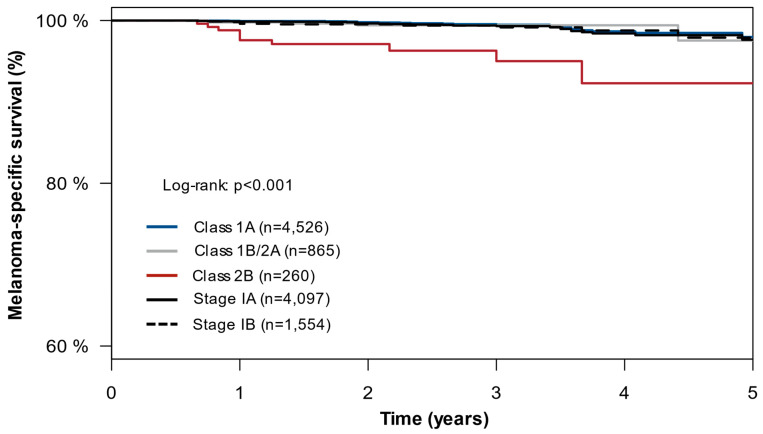
SEER cohort: melanoma-specific survival (MSS) risk stratification by the 31-GEP and AJCC in stage I tumors. KM curves showing 5-year MSS among patients in the SEER cohort based on 31-GEP result and AJCC stage.

We next analyzed patients in the SEER cohort to confirm the enhanced risk stratification of the 31-GEP relative to AJCC in a large, real-world, unselected population of patients clinically tested with the 31-GEP. Patient demographics are shown in Table 4. Patients with stage IA and stage IB CM had similar 5-year MSS rates (stage IA = 97.6%; 95% CI: 96.2–99.0% vs. stage IB = 97.9%, 95% CI: 95.9–99.9%; *p* = 0.600). When comparing the 31-GEP risk stratification to AJCC alone, patients with a Class 2B result had lower 5-year MSS (92.3%, 95% CI: 86.2–98.8%) than those with a Class 1A (98.0%, 95% CI: 96.7–99.2%) or Class 1B/2A 31-GEP result (97.5%, 95% CI: 93.9–100%) (*p* < 0.001) as well as those with stage IA or IB CM (Figure 2). Multivariable analysis showed that the 31-GEP Class 2B result was the only significant predictor of melanoma-specific mortality in stage I CM (HR = 9.23, *p* < 0.001).

## 4. Discussion

Patients with stage I CM generally have a good prognosis. However, AJCC staging is limited to MSS, and a subset of patients with stage I CM have tumors with high-risk gene expression indicating increased risk of recurrence and mortality. Indeed, recent studies found over 20% of recent melanoma deaths occurred in patients with thin tumors at diagnosis [4,27]. Another recent study found the most recent move from AJCC version 7 to version 8 reduced the discrimination of staging to predict RFS or overall survival of patients with stage I disease to that of a coin flip (AUC: 0.63 vs. 0.55) [6]. Furthermore, Garbe et al. found that MSS rates in a population-based German cohort were consistently less favorable than those published by AJCC [6,7]. Additional clinical tools have been developed to address shortcomings in standard AJCC staging, including nomograms that incorporate additional clinical and pathological factors; however, these were initially developed to predict SLNB positivity rather than stratifying survival outcomes. Importantly, they lack prospective or clinical utility data, and a recent report found they did not provide additional information beyond AJCC staging alone [13,14,28,29,30]. Another recent study investigated the risk stratification ability of a different GEP test (CP-GEP), which combines gene expression data for eight genes with age and Breslow thickness, to stratify patient risk of recurrence, and found that the CP-GEP did not stratify risk of recurrence better than AJCC in patients with stage I CM [31]. Thus, the CP-GEP test did not demonstrate additional value in stage I CM compared to AJCC staging alone.

In the present study, assessing almost 7000 patients with stage I CM, the 31-GEP test significantly stratified patient risk of recurrence or melanoma-specific death, consistently identifying patients with stage I disease with a higher risk of recurrence or death than predicted by AJCC alone, adding to the evidence that AJCC alone incorrectly classifies many tumors as low risk [7]. A major strength of this study was the large patient populations used to assess risk stratification. The combined cohort included patients from multiple sites, minimizing concerns that results may not apply to other patient populations [16,17,18,19,23]. Additionally, we analyzed over 5500 patients whose clinically tested 31-GEP results were linked with patient data from the SEER registries. The SEER Program covered approximately one-third of the United States population during the study period, providing a large, unselected cohort of patients to confirm stratification by the 31-GEP. Due to the substantial population size, the SEER cohort allows for clinically meaningful and statistically relevant conclusions regarding melanoma-specific mortality that are not always possible with smaller numbers of patients and events, particularly in stage I disease.

The 31-GEP test is an additional risk-stratification tool beyond the pathological assessment of the primary tumor biopsy. It is worth noting that NCCN and other clinical guidelines recommend considering an invasive SLNB surgical procedure if the likelihood of a positive node is greater than 5%, despite an overall complication rate over 10% [32,33]. The 31-GEP test is performed using tumor tissue from the primary tumor biopsy and thus has no potential for additional complications. It is worth noting that the proportion of Class 2B CM was ~5% in both the combined and the SEER cohorts, and the non-Class 1A (Class 1B/2A + 2B) was 20–23%, respectively.

The study had some limitations. Although using the SEER database allows for observations of a diverse, unselected population, the dataset limitations include underreported (chemotherapy and radiation) and incomplete information for some variables (e.g., Breslow thickness, and ulceration status for newer SEER registries). Additionally, SEER data do not include information about patient outcomes other than survival and cause of death, and the treatment data are limited to the first course of treatment (surgery, radiation and chemotherapy). Therefore, staging cannot be assessed using SEER data for some patients. Additionally, because SEER data do not include recurrence or metastasis data, we were only able to analyze MSS for this cohort. Among the combined cohort data, a portion of the previously published studies were retrospective, which brings the known limitations of those cohorts. However, given the large number of patients and the varied patient populations between the two cohorts, the conclusions here should generally apply to a wide patient population.

The 31-GEP test can significantly improve risk stratification compared to standard AJCC staging. Thus, the 31-GEP test can provide a personalized risk of recurrence, metastasis, or melanoma-specific mortality, allowing clinicians to provide risk-aligned treatment and surveillance management plans for their patients. Because patients with stage I CM are considered to have a low risk of poor outcomes, improved and personalized risk stratification methods can identify the high-risk patients among this low-risk population for better risk-aligned treatment and surveillance plans.

## 5. Conclusions

The data presented here confirm that the 31-GEP test has utility to address an unmet need in melanoma patient care—identifying patients classified as low risk by AJCC staging, but who have high-risk tumor biology and are more likely to experience poor outcomes. This study demonstrates that the 31-GEP added significant prognostic information beyond that of the clinicopathological factors included in standard AJCC8 CM staging. Thus, incorporating the 31-GEP into clinical practice may benefit patients by providing additional information that clinicians can use to make personalized, risk-aligned treatment and surveillance management plans.

## Figures and Tables

**Table 1:** Patient demographics and tumor characteristics—combined cohort.

Descriptor	Class 1A (n = 965)	Class 1B/2A (n = 226)	Class 2B (n = 70)	Combined (n = 1261)
Age (years), median (Range)	60 (13–91)	63 (19–95)	67 (25–88)	61 (13–95)
Sex				
Female	299 (31.0%)	52 (23.0%)	14 (20.0%)	365 (28.9%)
Male	331 (34.3%)	71 (31.4%)	19 (27.1%)	421 (33.4%)
Unknown	335 (34.7%)	103 (45.6%)	37 (52.9%)	475 (37.7%)
Tumor Location				
Extremity	453 (46.9%)	111 (49.1%)	31 (44.3%)	595 (47.2%)
Head and neck	192 (19.9%)	55 (24.3%)	20 (28.6%)	267 (21.2%)
Trunk	320 33.2%)	60 (26.5%)	19 (27.1%)	399 (31.6%)
Breslow thickness (mm), median (Range)	0.6 (0.08–2.03)	1.0 (0.1–2.0)	1.2 (0.18–2.03)	0.7 (0.08–2.03)
Ulceration				
No	883 (91.5%)	196 (86.7%)	59 (84.3%)	1138 (90.2%)
Yes	19 (2.0%)	11 (4.9%)	7 (10.0%)	37 (2.9%)
Unknown	63 (6.5%)	19 (8.4%)	4 (5.7%)	86 (6.8%)
Mitotic rate (1/mm^2^), median (Range)	0.0 (0.0–10.0)	1.0 (0.0–10.0)	1.0 (0.0–10.0)	0.0 (0.0–10.0)
Recurrence				
No	940 (97.4%)	205 (90.7%)	57 (81.4%)	1202 (95.3%)
Yes	25 (2.6%)	21 (9.3%)	13 (18.6%)	59 (4.7%)
Melanoma-specific death				
No	960 (99.5%)	222 (98.2%)	64 (91.4%)	1246 (98.8%)
Yes	5 (0.5%)	4 (1.8%)	6 (8.6%)	15 (1.2%)
T-stage				
T1a	581 (60.2%)	56 (24.8%)	11 (15.7%)	648 (51.4%)
T1b	206 (21.3%)	64 (28.3%)	16 (22.9%)	286 (22.7%)
T2a	178 (18.4%)	106 (46.9%)	43 (61.4%)	327 (25.9%)
AJCC 8th edition *				
Stage IA	713 (73.9%)	92 (40.7%)	20 (28.6%)	825 (65.4%)
Stage IB	252 (26.1%)	134 (59.3%)	50 (71.4%)	436 (34.6%)

Abbreviation: AJCC, American Joint Committee on Cancer; * AJCC stage was determined using Breslow thickness and ulceration status.

**Table 2:** Multivariable analyses.

Factor	Hazard Ratio (95% CI)	*p*-Value
RFS-Combined cohort		
31-GEP		
Class 1A	Reference	--
Class 1B/2A	2.63 (1.43–4.83)	0.002
Class 2B	5.16 (2.54–10.47)	<0.001
AJCC		
Stage IA	Reference	--
Stage IB	2.98 (1.64–5.40)	<0.001
MSS-Combined cohort		
31-GEP		
Class 1A	Reference	--
Class 1B/2A	2.35 (0.60–9.29)	0.223
Class 2B	11.08 (3.10–39.63)	<0.001
AJCC		
Stage IA	Reference	--
Stage IB	3.00 (0.87–10.37)	0.082
MSS-SEER cohort		
31-GEP		
Class 1A	Reference	--
Class 1B/2A	1.37 (0.50–3.71)	0.542
Class 2B	9.23 (4.23–20.18)	<0.001
AJCC		
Stage IA	Reference	--
Stage IB	0.82 (0.39–1.74)	0.609

**Table 3:** Likelihood ratio test.

Group	Likelihood Ratio	*p*-Value
RFS-Combined cohort		
31-GEP (Combined)	39.11	*p* < 0.001
AJCC (Combined)	32.05	*p* < 0.001
31-GEP added to AJCC (Combined)	52.99	*p* < 0.001
MSS-Combined cohort		
31-GEP (Combined)	19.29	*p* < 0.001
AJCC (Combined)	9.95	*p* = 0.002
31-GEP added to AJCC (Combined)	22.64	*p* < 0.001
MSS-SEER cohort		
31-GEP (SEER)	22.52	*p* < 0.001
AJCC (SEER)	0.20	*p* = 0.653
31-GEP added to AJCC (SEER)	22.79	*p* < 0.001

**Table 4:** Patient demographics and tumor characteristics—SEER cohort.

Descriptor	Class 1A (n = 4526)	Class 1B/2A (n = 865)	Class 2B (n = 260)	Combined (n = 5651)
Age (years), median (Range)	60 (18–90+)	64 (18–90+)	65 (22–90+)	61 (18–90+)
Sex				
Female	2089 (46.2%)	383 (44.3%)	96 (36.9%)	2568 (45.4%)
Male	2437 (53.8%)	482 (55.7%)	164 (63.1%)	3083 (54.6%)
Tumor Location				
Extremity	2054 (45.3%)	430 (49.7%)	141 (54.2%)	2625 (46.5%)
Head and neck	799 (17.6%)	209 (24.2%)	59 (22.7%)	1067 (18.9%)
Trunk	1648 (36.3%)	222 (25.7%)	60 (23.1%)	1930 (34.2%)
Not specified	25 (0.6%)	4 (0.5%)	0 (0.0%)	29 (0.5%)
Breslow thickness (mm), median (Range)	0.6 (0–2.0)	1.1 (0.1–2.0)	1.1 (0.1–2.0)	0.7 (0–2.0)
Ulceration				
No	3745 (82.7%)	667 (77.1%)	168 (64.6%)	4580 (81.0%)
Yes	92 (2.0%)	39 (4.5%)	30 (11.5%)	161 (2.8%)
Unknown	689 (15.2%)	159 (18.4%)	62 (23.8%)	910 (16.1%)
Mitotic rate (1/mm^2^), median (Range)	0 (0–11)	1 (0–11)	1 (0–11)	0 (0–11)
Melanoma-specific death				
No	4504 (99.5%)	860 (99.4%)	250 (96.2%)	5614 (99.3%)
Yes	22 (0.5%)	5 (0.6%)	10 (3.8%)	37 (0.7%)
T-stage				
T1a	2850 (63.0%)	199 (23.0%)	71 (27.3%)	3120 (55.2%)
T1b	936 (20.7%)	216 (25.0%)	52 (20.0%)	1204 (21.3%)
T2a	740 (16.3%)	450 (52.0%)	137 (52.7%)	1327 (23.5%)
AJCC 8th edition *				
Stage IA	3606 (79.7%)	375 (43.4%)	116 (44.6%)	4097 (72.5%)
Stage IB	920 (20.3%)	490 (56.6%)	144 (55.4%)	1554 (27.5%)

Abbreviation: AJCC, American Joint Committee on Cancer; * AJCC stage was determined using Breslow thickness and ulceration status.

## Data Availability

Patient data will not be made publicly available. SEER data are available from the National Cancer Institute SEER program as a specialized database following the established policies for data release.
